# Different Effects of Social Jetlag and Weekend Catch-Up Sleep on Well-Being of Adolescents According to the Actual Sleep Duration

**DOI:** 10.3390/ijerph20010574

**Published:** 2022-12-29

**Authors:** Lorenzo Tonetti, Alice Andreose, Valeria Bacaro, Martina Grimaldi, Vincenzo Natale, Elisabetta Crocetti

**Affiliations:** Department of Psychology “Renzo Canestrari”, University of Bologna, 40127 Bologna, Italy

**Keywords:** social jetlag, weekend catch-up sleep, actigraphy, adolescents, well-being, physical health

## Abstract

The aim of this study was to explore the potentially different associations between two common aspects of adolescents’ life, namely social jetlag and weekend catch-up sleep, with well-being and physical health, according to the actual sleep duration, i.e., <7 h and ≥7 h. To this end, 504 participants (42.1% males), with a mean age of 16.17 (standard deviation = 1.39), were examined in the this cross-sectional study. Participants were asked to wear the Micro Motionlogger Watch actigraph (Ambulatory Monitoring, Inc., Ardlsey, NY, USA) around their non-dominant wrist for seven consecutive days in order to objectively assess social jetlag and weekend catch-up sleep. Participants were also asked to fill in the Mental Health Continuum—Short Form for the assessment of subjective, social, and psychological well-being, as well as the SF-36 Health Survey for the perception of physical health. In adolescents sleeping less than 7 h, those experiencing weekend catch-up sleep longer than 120 min reported significantly lower subjective well-being compared to those with a weekend catch-up sleep duration between 0 and 59 min. These data pointed out the detrimental effect of long weekend catch-up sleep on self-reported well-being only in adolescents getting less than the recommended amount of sleep.

## 1. Introduction

According to a systematic review [1] which examined the time period between the year 1905 and the year 2008, a decrease of more than one hour of sleep per night was observed in children and adolescents. Moreover, it has been observed that over the time period between the year 1991 and the year 2012, the number of adolescents sleeping for at least seven hours per night has reduced, with the largest decrease taking place in 15-year-old adolescents [2]. Chronic sleep deprivation in teenagers has been associated with several negative outcomes, such as anxiety and school absenteeism [3].

In order to try to stem chronic sleep deprivation in adolescents, two different types of interventions have been conceived: on one hand, delaying the school start time [4,5] and, on the other, trying to soften the physiological phase delay of adolescents’ circadian system through morning light [6] or dawn simulation [7]. Both solutions have been tested and seem to have an effect on sleep duration; however, their feasibility in different social contexts is somewhat reduced. For example, delaying the school start time may lead to critical issues, such as management of the public transport timetable and socio-family rhythms. A third way to help adolescents to increase the overall amount of sleep they actually get is represented by psychoeducational interventions, mainly based on sleep hygiene rules [8]. However, the sleep/wake cycle of adolescents is still poorly understood. 

Adolescents, during the weekend, can carry out compensative behaviors for sleep deprivation. These behaviors are, in turn, affected by the adolescents’ desire to have fun. Two of the most important behaviors have been outlined: (1) delaying bedtime and get-up time during weekend days compared to weekdays, which is known as social jetlag [9]; (2) extending their sleep duration during weekend days compared to weekdays, which is referred to as weekend catch-up sleep [10].

*Social jetlag* is defined as a discrepancy between biological and social timing [9]. The term jetlag was used because this phenomenon looks like a marked shifting of several hours in bedtime and get-up time between weekdays and weekend days. As previously observed [11], due to a mismatch between the biological phase delay and the early school start time, the prevalence of social jetlag reaches its maximum value during adolescence. Social jetlag in adolescents has been associated with an increased body mass index [12], low academic performance [13], as well as anxious [14] and depressive [15] symptomatology, which is considered a matter of public health [16].

*Weekend catch-up sleep* can be described as an extended duration of sleep during the weekend days compared to the weekdays [10]. While Kang et al. [17] observed an association between weekend catch-up sleep duration in adolescents and suicide attempts, as well as self-injuries, subsequent studies highlighted that the effects of weekend catch-up sleep seem to be different according to its duration. In this regard, Zhang et al. [18] reported that an amount of weekend catch-up sleep longer than 2 h was significantly associated with higher risks of mood and behavior disorders, as well as suicidality, tobacco smoking, and lower perception of mental and physical health, compared to a condition of weekend catch-up sleep ranging in length between 1 min and 2 h. Moreover, this last duration of the weekend catch-up sleep was related to a lower risk of anxiety and behavior disorders, as well as tobacco smoking and perception of low physical health compared to the absence of weekend catch-up sleep. 

Comparing adolescents who get enough sleep (i.e., sleep duration ≥ 7 h [2]) with those who do not, the aim of the current study was to explore the potentially different associations between the different behaviors adopted during the weekend and their relationships with indices of psychophysical well-being. In more detail, the behavior adopted during the weekend was monitored via actigraphy in order to objectively identify those who engage in social jetlag, in weekend catch-up sleep, in both behaviors, and in neither of the two. Subjective indices of psychophysical well-being (in terms of subjective, social, and psychological well-being, as well as physical health perception) were also collected. Therefore, the ultimate aim of our study was to highlight if there is a behavior that qualifies as the most protective sleep-recovery strategy for the psychophysical well-being of adolescents. The promotion of such an evidence-based strategy in psychoeducational interventions could have relevant implications for the everyday life of adolescents.

## 2. Materials and Methods

### 2.1. Participants

An overall number of 504 participants (42.1% of males), with a mean age of 16.17 (standard deviation = 1.39, range = 14.35–18.87), were examined in the current study. Participants were attending either the first year (50.7%) or the third year (49.3%) of secondary high school. 

### 2.2. Actigraphy

The actigraph model Micro Motionlogger Watch (Ambulatory Monitoring, Inc., Ardsley, NY, USA) was used in the current study. The device was equipped with a triaxial accelerometer with a sensitivity of 0.01 g, while the filters and sampling frequency were set at 2–3 Hz and 32 Hz, respectively. Actigraphs were initialized through the Motionlogger Watchware software (Ambulatory Monitoring, Inc., Ardsley, NY, USA) in zero crossing mode to collect data in 1 min epochs, while actigraphic records were analyzed through the software Action W2 (Ambulatory Monitoring, Inc., Ardsley, NY, USA) using the algorithms validated by Cole and Kripke [19] and Cole et al. [20]. 

### 2.3. Actigraphic Social Jetlag and Weekend Catch-Up Sleep

Social jetlag was computed as the difference in the midpoint of sleep (i.e., the time of day that splits in half the time spent in bed) recorded during weekend days and school days. The length of the social jetlag is expressed in hours and minutes, with positive values pointing to a delayed midpoint of sleep during weekend days compared to school days. Participants were classified according to the following length of social jetlag: <60 min, 60–119 min, and ≥120 min. We selected those categories based on the criterion of the length of social jetlag, in line with the classification by Islam et al. [21]. We observed that 27.8% of participants were in the lowest duration category, 38.3% were in the middle duration category, and 33.9% were in the highest duration category.

The weekend catch-up sleep was calculated as the difference in the total sleep time (i.e., the sum in minutes of all the sleep epochs between sleep onset and get-up time) between weekend days and school days. Therefore, a positive value means a higher duration of total sleep time during weekend days compared to school days. Participants were grouped according to the following length of weekend catch-up sleep: <0 min; 0–59 min; 60–119 min; ≥120 min. We selected those categories according to the criterion of the frequency distribution, keeping the quartiles as a reference, with 22.1% in the first, 27% in the second, 25.4% in the third, and 25.5% in the fourth. This classification was also overall in line with that adopted by Kim et al. [22]. 

### 2.4. Subjective Measures of Well-Being and Physical Health Perception

Adolescents self-reported their well-being through the Italian version [23] of the Mental Health Continuum–Short Form (MHC–SF) [24]. The MHC-SF is composed of 14 items on a 6-level Likert scale with answers ranging from “never” to “every day”. Out of the 14 items, 3 are used to compute subjective well-being (e.g., “During the last four months, how often did you feel satisfied with life?”), five for social well-being (e.g., “During the last four months, how often did you feel that you belonged to a community (like a social group, or your neighborhood)?”, and six for psychological well-being (e.g., “During the last four months, how often did you feel that you liked most parts of your personality?”). Subjective, social, and psychological well-being are computed by summing up the scores of the corresponding items, with higher scores pointing to higher well-being.

Physical health perception was self-reported through the Italian version [25] of the subscale “General Health (GH)” from the SF-36 Health Survey [26]. The subscale is composed of four items on a 5-level Likert scale, ranging from “poor” to “excellent” for the first item (i.e., “In general, would you say your health is…”) and from “completely untrue” to “completely true” for the remaining three items (e.g., “My health is excellent”). Physical health perception is computed by summing up the scores of the four items, with higher scores pointing to higher physical health perception. 

### 2.5. Procedure

Participants were drawn from the ongoing ERC-Consolidator project IDENTITIES “Managing identities in diverse societies: A developmental intergroup perspective with adolescents” project. This longitudinal research involved 1st and 3rd-year students (at the first assessment) from several high schools located in the northern part of Italy (i.e., the Emilia-Romagna region). Schools were selected through a stratified (by school track and level of urbanization) randomized method, and principals were approached to present the project. Active consent from parents was obtained prior to the children’s participation. Active consent was also obtained from adolescents of age, while their underage peers provided their assent to participate in the project. Participation in the study was voluntary, and students were informed that they could withdraw their consent at any time. 

The longitudinal ongoing project started in 2022 and includes different assessments. For the present study, data from the wave collected April–May 2022 have been used. 

Participants were invited to wear the actigraph Micro Motionlogger Watch (Ambulatory Monitoring, Inc., Ardsley, NY, USA) around their non-dominant wrist for seven consecutive days, for 24 h per day. Moreover, they were instructed to push the event-marker button of the device to signal their bedtime and the get-up time. At the end of the actigraphic recording, participants filled in the questionnaires during class hours. 

Participants were included in the final database for statistical analyses only if, for the actigraphic recording, they had at least three valid nights during school days and one valid night during the weekend. According to the criteria put forward by Keyes et al. [2], participants were then split into two groups based on sleep duration: (1) those who slept enough, i.e., ≥7 h; (2) those who did not get enough sleep, i.e., <7 h. 

### 2.6. Statistical Analyses

Pearson’s correlation analyses were performed separately for each sleep duration group, examining the duration (in minutes) of social jetlag and weekend catch-up sleep from one side and the scores of subjective, social, and psychological well-being, as well as physical health perception on the other. 

One-way analysis of variance (ANOVA) was performed with the sleep duration group (two levels) as the between-subjects factor and subjective, social, and psychological well-being, and physical health perception scores as the dependent variables.

Moreover, we verified the distribution of participants, classified in categories according to the duration of social jetlag and weekend catch-up sleep, among the sleep duration group through a set of chi-squared tests.

Finally, separately for each sleep duration group, we independently assessed the effects of the social jetlag and weekend catch-up sleep categories as independent variables on the scores of subjective, social, and psychological well-being besides physical health perception as the dependent variable, through a set of one-way ANOVAs. In case of significant effects, the HSD Tukey post hoc test was used, with the significance level set to *p* < *0*.05.

## 3. Results

As reported in Table 1, significant correlations were observed only in adolescents sleeping less than 7 h. In particular, a higher duration of weekend catch-up sleep was significantly associated with lower subjective-, social-, and psychological well-being, as well as a reduced perception of physical health.

As shown in Table 2, adolescents did not significantly differ in subjective, social, and psychological well-being or perception of physical health according to the duration of sleep (i.e., <7 h or ≥7 h).

As highlighted in Table 3, the distribution of adolescents sleeping < 7 h or ≥ 7 h was not significantly different among the categories based on the duration of the social jetlag (χ^2^_2_ = 2.53; *p* = 0.28). 

On the contrary, a significantly different distribution of adolescents, based on their sleep duration, was observed among the categories of weekend catch-up sleep (χ^2^ = 32.04; *p* < 0.001). In particular, among adolescents sleeping less than 7 h, it was observed that a higher percentage of participants presented a weekend catch-up sleep equal to or longer than 120 min compared to the adolescents sleeping ≥ 7 h (Table 4). 

As shown in Table 5, within both groups of adolescents sleeping less than 7 h and equal to or higher than 7 h per night, no significant effects of the social jetlag categories on the outcomes of well-being and physical health perception were observed.

With reference to the group of adolescents sleeping for less than 7 h, we observed a significant effect of the weekend catch-up sleep categories on subjective well-being (Table 6). Performing the HSD Tukey post hoc test, adolescents with an amount of weekend catch-up sleep equal to or higher than 120 min reported a significantly lower level of subjective well-being than those showing a duration of the weekend catch-up sleep between 0 and 59 min (*p* < *0*.05), while the other post hoc comparisons did not reach significance. As regards the group of adolescents sleeping 7 or more hours per night, no significant effects of the weekend catch-up sleep categories were observed in the outcomes of this study (Table 6).

## 4. Discussion

The main goal of the current study was to explore the potentially different effects of two frequent behaviors adopted by adolescents, namely social jetlag and weekend catch-up sleep, on psychophysical well-being. In more detail, we were interested in determining whether these relationships were different between adolescents getting enough sleep and those who were chronically sleep deprived. 

The main result of the current study is that no behavior adopted during the weekend seems to be related to the well-being of adolescents” with “no behavior per se, adopted during the weekend, seems to be related to the well-being of adolescents. On the contrary, it seems that only the interaction between the weekend behavior and the actual sleep duration was able to modulate adolescents’ adjustment. In more detail, it was observed that longer weekend catch-up sleep was significantly associated with lower well-being only in the group of sleep-deprived adolescents (Table 1), therefore pointing out that the well-being of adolescents depends on the starting condition (namely, the sleep deprivation) that interacts with the compensatory weekend behavior (that is, the weekend catch-up sleep).

Moreover, it is also interesting to underline that, within the group of sleep-deprived adolescents, only a duration of weekend-catch-up sleep higher than 120 min was significantly related to lower well-being, compared to a duration between 0 and 59 min (Table 6). These data highlight that the negative effects of weekend catch-up sleep are not independent of its duration while, on the contrary, they seem to appear only when its length is over the cut-off value of 2 h. It is noteworthy that in the current study, among the group of adolescents sleeping less than 7 h, more than one-third (i.e., 34.1%) of the participants presented an amount of weekend catch-up sleep higher than the critical cut-off value of 2 h (Table 4), which could be interpreted as a compensative behavior to the starting condition, i.e., the sleep deprivation.

Based on our data, therefore, it seems that increasing adolescents’ sleep duration may have a protective effect on their well-being. To increase the duration of sleep, a specific intervention could be assessed and, in case of positive outcomes, promoted. More in detail, a short daily restorative nap [27,28] during schooldays that on one hand, may soften the sleep deprivation with a small recovery period and, on the other, may avoid the weekend sleep binge, could be introduced. In other words, based on the data of our study, i.e., negative effects of weekend catch-up sleep longer than 2 h on well-being in sleep-deprived adolescents, a short systematic daily nap during schooldays may be implemented, to maintain a period of weekend catch-up sleep no longer than 1 h. In case of positive outcomes on adolescents’ well-being, this strategy may be promoted as a sleep hygiene rule [8] within psychoeducational interventions.

The present study has some strengths; the assessment of sleep behaviors was objective (i.e., through actigraphy) and ecological (i.e., in the everyday life environment), besides being carried out in a large sample of adolescents. However, this study is not free from limitations; indeed, an accurate anamnesis, in order to exclude common pediatric diseases affecting the sleep/wave cycle as adenoid hypertrophy, tonsillar hypertrophy, allergic rhinitis or chronic rhinosinusitis, was not carried out. Moreover, since this is a study carried out in the real world, it was not possible to assess and control for potential confounding factors such as hormonal changes and pathological and psychophysical diseases. Furthermore, only the well-being of adolescents was examined as an outcome measure of social jetlag and weekend catch-up sleep, without taking into account the cognitive impact of both strategies—in other words, the academic performance of adolescents. Previous studies have reported an effect of social jetlag [13] and weekend catch-up sleep [29], although most studies measured these two behaviors subjectively. As highlighted in the systematic review by Sun and colleagues [29], “Future research should consider adopting longitudinal and experimental designs, and the use of objective sleep measures to further examine the impacts of weekday-to-weekend sleep differences on mental and physical health in the young population and to gain more insight into the mechanisms underlying their associations” (page 51). We believe that the current ongoing longitudinal study may have the potential, in the future, to address this specific point, taking into account the answers of participants to a question about the current grade-point average (GPA). Moreover, we should keep in mind that we have taken just one picture of the behavior of adolescents for one week and their perception of well-being during that week, i.e., we have observed a sort of immediate-phasic effect of the behavior adopted by adolescents on their well-being. Therefore, we are not currently able to answer this question: are there any effects of adolescents’ behavior on well-being in the short-, medium-, and long-term? However, the current study might have the potential to answer this question properly. Indeed, this ongoing longitudinal study will examine adolescents over time, potentially detecting the effects in the short (e.g., 4 months), medium (e.g., 1 year), and long term (e.g., 2 years or more). If feasible, the length of actigraphic monitoring should be extended to at least two weeks [30]. Finally, it will also be interesting to examine the potential role played by the socio-economic status of the families [31] and other significant contextual factors, such as the school track in which adolescents are enrolled.

## 5. Conclusions

The aim of the present study was to explore the potentially different associations, depending on the length of sleep, between two frequent behaviors adopted by adolescents, i.e., social jetlag and weekend catch-up sleep, and well-being. The main result is that, among sleep-deprived adolescents, weekend catch-up sleep longer than 120 min was significantly associated with lower well-being compared to those with a weekend catch up-sleep duration between 0 and 59 min. The take-home message of the current study is that no specific behavior per se was associated with well-being, but it seems that a sort of interaction between the starting condition—namely sleep deprivation—and one behavior—that is, weekend catch-up sleep—may play the primary role in the modulation of adolescents’ well-being. 

## Figures and Tables

**Table 1:** Pearson’s correlation values (with the *p*-value within brackets) between social jetlag and weekend catch-up sleep on one side and subjective, social, and psychological well-being, and physical health perception on the other, separately for the sleep duration group. Significant correlations are highlighted in bold.

	Adolescents Sleeping < 7 h			
	Subjective Well-Being	Social Well-Being	Psychological Well-Being	Physical Health Perception
Social jetlag	−0.011 (*p* = 0.852)	0.092 (*p* = 0.117)	0.038 (*p* = 0.518)	0.037 (*p* = 0.530)
Weekend catch-up sleep	**−0.194** **(*p* < 0.001)**	**−0.147** **(*p* < 0.05)**	**−0.166** **(*p* < 0.01)**	**−0.124** **(*p* < 0.05)**
	**Adolescents sleeping ≥ 7 h**			
	**Subjective ** **Well-Being**	**Social Well-Being**	**Psychological** **Well-Being**	**Physical Health** **Perception**
Social jetlag	−0.029 (*p* = 0.679)	0.010 (*p* = 0.881)	−0.046 (*p* = 0.518)	−0.049 (*p* = 0.481)
Weekend catch-up sleep	−0.030 (*p* = 0.672)	−0.015 (*p* = 0.830)	−0.016 (*p* = 0.816)	−0.012 (*p* = 0.865)

**Table 2:** Means and standard deviations of the subjective, social, and psychological well-being as well as physical health perception by sleep duration group. Statistics are also reported.

	Adolescents Sleeping < 7 h	Adolescents Sleeping ≥ 7 h	Statistics
Subjective well-being	4.06 ± 1.09	4.08 ± 1.16	F_1,494_ = 0.03; *p* = 0.86
Social well-being	3.11 ± 1.26	3.12 ± 1.18	F_1,495_ = 0.01; *p* = 0.94
Psychological well-being	3.87 ± 1.13	3.95 ± 1.14	F_1,490_ = 0.50; *p* = 0.48
Physical health perception	3.81 ± 0.67	3.81 ± 0.64	F_1,502_ = 0.01; *p* = 0.93

**Table 3:** Distribution of participants sleeping < 7 h or ≥ 7 h among the categories based on the duration of social jetlag. Frequency and percentage are shown.

	Adolescents Sleeping < 7 h		Adolescents Sleeping ≥ 7 h	
	Frequency	Percentage	Frequency	Percentage
<60 min	81	25.3	70	31.5
60–119 min	127	39.7	80	36
≥120 min	112	35	72	32.4

**Table 4:** Distribution of participants sleeping < 7 h or ≥ 7 h among the categories based on the duration of weekend catch-up sleep. Frequency and percentage are shown.

	Adolescents Sleeping < 7 h		Adolescents Sleeping ≥ 7 h	
	Frequency	Percentage	Frequency	Percentage
<0 min	57	17.8	63	28.4
0–59 min	79	24.7	66	29.7
60–119 min	75	23.4	64	28.8
≥120 min	109	34.1	29	13.1

**Table 5:** Means and standard deviations of subjective, social, and psychological well-being as well as physical health perception according to the extent of the social jetlag, separately for sleep duration group. Statistics are also reported.

		Social Jetlag	Adolescents Sleeping < 7 h	
	<60 min	60–119 min	>120 min	Statistics
Subjective well-being	3.99 ± 0.99	4.16 ± 1.10	3.99 ± 1.15	F_2,288_ = 0.85; *p* = 0.43
Social well-being	2.94 ± 1.19	3.15 ± 1.27	3.19 ± 1.29	F_2,288_ = 0.97; *p* = 0.38
Psychological well-being	3.70 ± 1.05	4.01 ± 1.12	3.84 ± 1.19	F_2,285_ = 1.8; *p* = 0.17
Physical health perception	3.88 ± 0.67	3.79 ± 0.69	3.79 ± 0.64	F_2,294_ = 0.49; *p* = 0.62
		Social jetlag		Adolescents sleeping ≥ 7 h
	<60 min	60–119 min	120 min	Statistics
Subjective well-being	4.14 ± 1.22	4.03 ± 1.22	4.07 ± 1.03	F_2,202_ = 0.17; *p* = 0.84
Social well-being	3.18 ± 1.28	2.98 ± 1.16	3.21 ± 1.10	F_2,203_ = 0.79; *p* = 0.46
Psychological well-being	4.02 ± 1.24	3.93 ± 1.13	3.90 ± 1.07	F_2,201_ = 0.20; *p* = 0.82
Physical health perception	3.85 ± 0.73	3.77 ± 0.60	3.81 ± 0.61	F_2,204_ = 0.23; *p* = 0.79

**Table 6:** Means and standard deviations of subjective, social, and psychological well-being as well as physical health perception according to the extent of the weekend catch-up sleep, separately for sleep duration group. Statistics are also reported, with significant effects in bold.

		Weekend Catch-Up Sleep		Adolescents Sleeping < 7 h	
	<0 min	0–59 min	60–119 min	≥120 min	Statistics
Subjective well-being	**4.31 ± 0.88**	**4.31 ± 1.11**	**3.83 ± 1.15**	**3.91 ± 1.08**	**F_3,287_ = 3.94; *p* < 0.01**
Social well-being	3.40 ± 1.10	3.26 ± 1.33	3 ± 1.32	2.94 ± 1.22	F_3,287_ = 1.98; *p* = 0.12
Psychological well-being	4.07 ± 0.88	4.09 ± 1.09	3.78 ± 1.08	3.70 ± 1.28	F_2,284_ = 2.41; *p* = 0.07
Physical health perception	3.86 ± 0.76	3.84 ± 0.66	3.92 ± 0.65	3.70 ± 0.63	F_2,293_ = 1.74; *p* = 0.16
		Weekend catch-up sleep			Adolescents sleeping ≥ 7 h
	<0 min	0–59 min	60–119 min	≥120 min	Statistics
Subjective well-being	4.14 ± 1.13	3.96 ± 1.29	4.19 ± 1.07	3.97 ± 1.08	F_3,201_ = 0.53; *p* = 0.70
Social well-being	3.11 ± 1.10	3.03 ± 1.27	3.24 ± 1.14	3.10 ± 1.27	F_3,202_ = 0.30; *p* = 0.82
Psychological well-being	3.98 ± 1.10	3.81 ± 1.26	4.11 ± 1.10	3.85 ± 1.05	F_3,200_ = 0.77; *p* = 0.51
Physical health perception	3.76 ± 0.70	3.88 ± 0.68	3.81 ± 0.58	3.73 ± 0.56	F_3,203_ = 0.48; *p* = 0.70

Significant effect is highlighted in bold.

## Data Availability

The data are not publicly available and cannot be shared due to ethical issues.
